# Distemper in the Dog1Presented by title before the Thirty-eighth Annual Meeting of the American Veterinary Medical Association.

**Published:** 1901-11

**Authors:** William McEachran

**Affiliations:** Windsor, Ont.


### DISTEMPER IN THE DOG.[1]

By William McEachran, M.D., C.M., V.S.,

WINDSOR, ONT.

The treatment of the diseases of the dog is a subject of the very greatest importance to the average practitioner to-day, owing to the large number of valuable dogs to be found everywhere, and their increasing value as hunting, show, and pet dogs. Having always taken a lively interest in the subject, and observing its absence on last year's programme, is my excuse for presenting it at this meeting.

The name distemper is the common one for this affection, but I think it is too general, and so prefer the name catarrhal fever, as it conveys a better idea of the character of the disease in question. It is a specific fever, due to a germ (undetermined). The germ, entering the blood by way of the mouth and stomach or air-

[1] Presented by title before the Thirty-eighth Annual Meeting of the American Veterinary Medical Association.

passages, manifests itself at first by fever, the point of the dog's nose giving the first indication, being dry and hot. A rise in temperature takes place, followed by irritation of the mucous membranes of the body, especially the nose and eyes, and followed by serious affections of the lungs, bowels, and nervous system. It is highly contagious and infectious, and large numbers of dogs under one year old are lost annually in all parts of the world, so widespread is it at the present time.

The cause of the disease is specific, but many predisposing causes may also be mentioned which modify its symptoms to a greater or less degree. The age of the dog has an influence, as it is found that young dogs are most subject to it and suffer most from its attacks. Unsanitary surroundings and poor care render dogs more liable to contract the disease in a severe form. Overfeeding with meat, by producing alimentary derangements, increases the severity of the disease once established. Overcrowding in damp, dark kennels in the spring and fall renders them more liable to contract it by lowering their vitality. But all these conditions taken together will not produce distemper; the germ of the disease must be present.

*Symptoms.* There are two forms of distemper—benign and malignant. In the first form there is a mild attack, in which we find a slight watery discharge from the nostrils, accompanied by sneezing or a slight cough; this may last a day or two, when the eyes are observed to show a slight watery discharge and the nose is dry and hot. The discharges soon become mucopurulent and sticky, so much so as to close the eyelids in the mornings. The appetite is more or less diminished, and there is great thirst; the temperature, if taken, is found to be elevated (100° to 103° F.). These may be the only symptoms, the dog running around much as usual, and they may last for three or four days only; when the fever subsides the appetite returns and the discharges from the eyes and nose disappear, and in from a week to ten days the animal appears to be as well as ever.

*Malignant.* In the malignant form of distemper the sneezing and coughing increase rapidly in severity, occurring in paroxysms. The cough is at first dry and husky, the dog acting as if he had a bone in his throat and is anxious to get rid of it; later there is a profuse discharge of saliva and much slobbering. There is a discharge from both the eyes and the nose, which soon becomes thick and purulent, leaving dirty crusts on the cheeks and around the nostrils. The discharge adheres to the eyelashes and hair on the cheeks and eyebrows, and removes it, leaving the appearance referred to as *spectacles*. The eyes are dull and heavy, the conjunctiva becoming a bright red, in most cases in a few days, usually about the fourth or fifth, a bluish, cloudy scum is observed, and near the centre a small round ulcer may be seen on the cornea. This has a tendency to spread, producing blindness in one or both eyes. As the disease progresses the ulceration may spread, or an abscess may form beneath, which may empty into the chambers or externally ; in either case the sight of the eye is completely lost. The appetite may not be interfered with for the first four or five days, but then it diminishes, and he refuses food and becomes weak and staggering in his gait, and lies down a great deal. The temperature is elevated to 104° F., the pulse is rapid and weak, and the discharges are profuse. From the sixth to the tenth day the condition of the bowels will attract attention. He is observed to be "tucked up ;" he may at first become constipated, with great tenesmus, but later this may change to diarrhœa or even to dysentery, when the discharge is tinged with blood. About the tenth day, especially if the weather be cold and changeable, the catarrhal symptoms progress and the tendency is to descend to the bronchial tubes and lung tissue, so that he may have a more or less severe attack of bronchitis or bronchopneumonia, indicated by the symptoms of these affections. At this stage (tenth to the twelfth day) the disease may remain stationary for a few days, and then progress unfavorably, or recovery may set in, the symptoms gradually subsiding, or it may vary in intensity, the appetite returning and again disappearing. The symptoms are more favorable one day and less so the next, and if properly treated now recovery may take place in a few weeks. Should it progress unfavorably the symptoms become more severe, the breathing rapid and labored, the debility very great ; there is observed a peculiar, anxious look on the face, the eyes are sunken in the orbits, and partly hidden by the *membrana nictitans;* the discharges are now (about the third week) more offensive and brownish-colored, and often streaked with blood. The breathing is peculiar, the animal inflating the cheeks at each expiration, and drawing them in at each inspiration ; the skin becomes dry and unhealthy looking ; a most disagreeable odor is given off, which is peculiar to this disease of the dog. Recovery is very rare at this stage, death being due to exhaustion or to suffocation from occlusion of the bronchial tubes and lungs.

Gastritis, diarrhœa, jaundice, and nervous affections may or may not complicate the ordinary case of distemper. The nervous affections are, I think, worthy of most attention, being least understood. Two forms occur—one as *chorea*, and the other as *epileptic fits.*

*Chorea.* In many parts of this continent chorea is very much dreaded and is looked on by breeders as a separate disease, and many valuable puppies showing these symptoms are annually lost. It is, however, a result of the action of the virus of catarrhal fever. It may appear in an acute form early in the disease, but usually when the symptoms of catarrh, especially of the bronchi, have been well established, or diarrhœa has complicated the other symptoms and great debility is manifested. It is observed as "a continuous succession of entirely involuntary clonic contractions;[1] these involve the whole body, prevent the animal from resting, and almost hinder it from eating or drinking. In many cases the animal lies perfectly helpless, moaning or yelling continuously." After a day or two of intense suffering he dies.

In the chronic form the symptoms are not so severe. Usually only one limb—a fore one—is at first affected, or the head, eyelash, ear, or a hind leg, or even all of one side shows the peculiar, regular, rhythmic contractions. These are more or less under control, the tendency is to progress in severity, but when the other symptoms of distemper subside or disappear these will generally remain. The general health of the dog is then little affected. The symptoms may be observed in many cases, even in sleep, although in others sleep brings complete quiet. They are most marked if you excite the animal in any way.

*Epileptic Fits.* The epileptic fits are always acute and indicate a serious condition of the dog. They are usually progressive, one following another unless relieved. The seizure comes on suddenly; he may be lively and playing about and may eat fairly well. It may be just after eating that he is observed to have some spasmodic contractions of the muscles of the head or of some other part of the body; he champs the lower jaw and foams at the mouth; he rolls his eyes and has a wild look in them; he will stand trembling, or staggers and falls to the ground unconscious, and lies struggling and pawing the air. In other cases he rushes about, barking and crying in a moaning way, going on until he falls or meets some obstacle. The fit may last a variable length of time, and he may get up showing a dazed condition, but seems to recover

[1] Fleming's Sanitary Science and Police.

more or less for a time. The next day he will have a return of the same, and gradually they increase in frequency and severity until great prostration and paralysis set in, when they subside, becoming weaker until, total paralysis setting in, he dies, or he may live paralyzed on one side.

A peculiarity I have observed with regard to the nervous affections above spoken of is the large number of cases presenting the symptoms of chorea or epilepsy which I have met in which there has been an absence of any other symptoms of distemper, although other cases have existed in the same kennel having all the ordinary symptoms with no nervous conditions. This I have observed particularly among cocker spaniels.

The pathological changes are such as we would expect from the symptoms and complications. Evidence of catarrhal inflammation of the eyes, air-passages, and alimentary tract and liver, congestion and effusion into the cavities of the brain and spinal canal will be found post-mortem.

The diagnosis of catarrhal fever is not, as a rule, a difficult matter for the practitioner. Rabies is the only disease which presents serious difficulty, but a comparison of the symptoms in the two affections will lead easily to a correct diagnosis.

*Treatment.* In the treatment of distemper the stage of the disease has to be taken into consideration. In the early stage the dog should be properly housed; if in cold weather, in a comfortable kennel, though not too warm. His food should be, as a rule, light and easily digested. I do not, however, change the food to which he has been accustomed, provided his appetite continues good. Should his appetite cease to any extent, I try him with a variety of foods—oatmeal, bread and milk, beef-tea, and bread, minced meat (raw or cooked), or, in fact, anything he can be induced to take. Should it fail entirely he should be fed with beef-tea, milk, egg-nog, etc.

In some mild cases it would appear as if the disease were cut short by treatment, but I am not of the opinion that distemper can be cut short by any remedies. The affection if in a mild form can be relieved, but it will take a course of one or two weeks. In mild cases the fever may be controlled and reduced by giving various antifebrifuges, quinine in one to five-grain doses, according to size and age of the dog. I have found aconite a most useful remedy. For the catarrhal symptoms, chlorate of potash three times a day is probably the best; a sedative may be given if the cough is severe. The bowels, if constipated, should have a laxative, such as syrup rhamni, or a mixture of equal parts of syrup rhamni and castor oil. Should diarrhœa be present the administration of tr. opii and creta preparata are indicated. Camphor I have also found useful in full doses. The bronchial affection (besides the general treatment) is best controlled by the application of a stimulating liniment to the sides, followed by linseed poultices or hot-water bags. It is best to remove the hair. If laryngitis be present a stimulating liniment is beneficial.

The eyes should be carefully attended to from the beginning. They should be bathed twice a day and a two or three-grain solution of argentum nitrate or a wash of boracic acid applied by brush or dropper. More serious affections of the eyes will require surgical treatment, even to the removal of the eye.

The nervous symptoms are the most difficult to treat. I have succeeded in cutting them short in a large number of cases by giving a mixture of chloral hydrate and potassium bromide in full doses, and continue while the nervous symptoms last. In chronic cases very little can be done besides attending to the general health; in many cases it seems to disappear spontaneously. In a few cases I have had good results from prolonged administration of potassium iodide. In all cases where the prostration is great I use a stimulant of some kind, preferring port or sherry wine.

During convalescence tonics and stimulants are indicated in connection with general good care. In large kennels, of course, general measures of isolation and disinfection should be carried out.

